# Malignant phosphaturic mesenchymal tumor-ossifying fibroma-like subtype: a case report and literature review

**DOI:** 10.1186/s12891-021-04558-1

**Published:** 2021-08-10

**Authors:** Hongyu Qin, Hao Zeng, Hao Li, Shuangshuang Yuan, Jinsong Yang

**Affiliations:** The First Affiliated Hospital of Guangxi Medical University, Shuangyong Rd, Qingxiu District, Nanning, GuangXi China

**Keywords:** Ossifying fibroma-like subtype, Phosphaturic mesenchymal tumor, Tumor resection

## Abstract

**Background:**

A phosphaturic mesenchymal tumor (PMT) is classified into four histological subtypes: mixed connective tissue, osteoblast-like, non-ossifying fibroma-like, and ossifying fibroma-like. The ossifying fibroma-like subtype being extremely rare. Most PMTs are benign, with a minimal number becoming malignant after recurrence. In this study, we report a case of recurrence and malignant transformation of PMT-ossifying fibroma-like subtype in the left hip bone.

**Case presentation:**

Here, we report the clinical manifestations, histology, pathological features, and treatment of a 57-year-old Chinese woman with a recurrent and malignant ossifying fibroma-like subtype PMT of the left iliac bone. The tumor was first discovered 3 years ago when the patient underwent surgery to remove the tumor. Precisely 2 years and 6 months after the operation, the pain in the left hip reappeared. After 6 months, the patient went to our hospital for treatment. After the tumor resection, the postoperative symptoms improved significantly, and the serum alkaline phosphatase level returned to normal. Based on clinical manifestations, evaluation of serum biochemical indicators, X-ray examination, computerized tomography scan of the pelvis, and histopathological examination of the two operations, the patient was finally diagnosed with a recurring and malignant transformation of the left iliac bone phosphaturic mesenchymal tumor-ossifying fibroma-like subtype. No tumor recurrence was found during the follow-up 15 months after the operation.

**Conclusions:**

This case increases the awareness of a rare malignant subtype of PMT and provides a valuable reference for the diagnosis of this disease.

## Background

A phosphaturic mesenchymal tumor (PMT) is a rare abnormal growth that is classified into four histological subtypes: mixed connective tissue, osteoblast-like, non-ossifying fibroma-like, and ossifying fibroma-like [1]. The mixed connective tissue subtype is the most common [2], with the ossifying fibroma-like subtype being extremely rare. Most PMTs are benign, with a minimal number becoming malignant after recurrence [3, 4]. In this study, we report a case of recurrence and malignant transformation of PMT-ossifying fibroma-like subtype in the left hip bone.

## Case presentation

A 57-year-old Chinese woman came to our hospital for treatment. The patient indicated that the pain and soreness in the left hip had lasted for 6 months, accompanied by swelling in the left groin area, and gradual difficulty in walking. These symptoms could be relieved via rest and painkillers were needed at times. The patient’s personal and family history was not unusual, but the previous medical history showed that the patient had also experienced pain in the left hip 3 years before admission. At that time, she was diagnosed with a left iliac bone tumor in her hometown hospital and underwent tumor resection After treatment, the symptoms were relieved.

Physical examination showed that the patient’s left groin was swollen, and an old surgical scar about 8 cm in length was visible on the left hip. A mass of about 10.0 8.0 cm in size could be palpated. The boundary was unclear, with no tenderness, medium texture, normal superficial skin temperature, no varicose vessels and skin ulcers, normal limb sensation, normal muscle strength of the limbs, unrestricted activity, and no abnormalities in the neurological examination.

Upon admission, an evaluation of the serum biochemical indicators indicated high serum alkaline phosphatase (211 U/L) and normal serum calcium (2.510 mmol/L) and phosphorus (1.150 mmol/L) levels. The serum levels of the following tumor markers were normal: Carbohydrate antigen 199, carbohydrate antigen 125, carcinoembryonic antigen, and alpha-fetoprotein. No results were available for serum fibroblast growth factor (FGF-23), serum 1-α,25-dihydroxyvitamin D3 (1,25-(OH)-D3) levels, or urine phosphate.

**Fig. 1:**
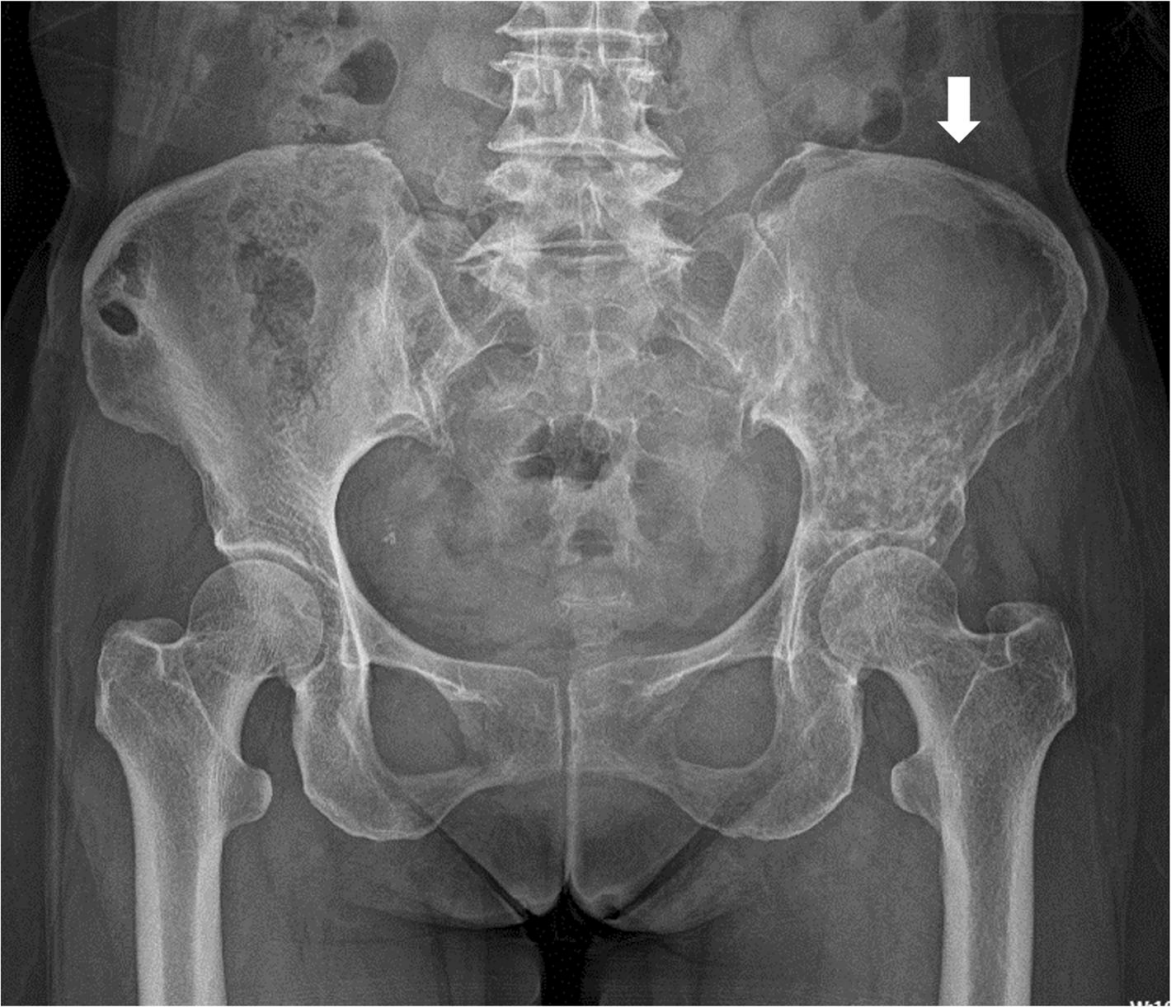
X-ray of pelvis. A circular low-density defect area of the left iliac bone, with clear boundaries and no hardening at the edges. The size is about 7.2 × 5.7 cm (shown by white arrows)

X-ray of the pelvis (Fig. 1) indicated a round, low-density bone defect area of the left iliac bone, with clear boundaries and no hardening at the edges. The size was about 7.2 × 5.7 cm. Uneven increase in bone density in the rest of the left iliac bone involved the left acetabulum.

**Fig. 2:**
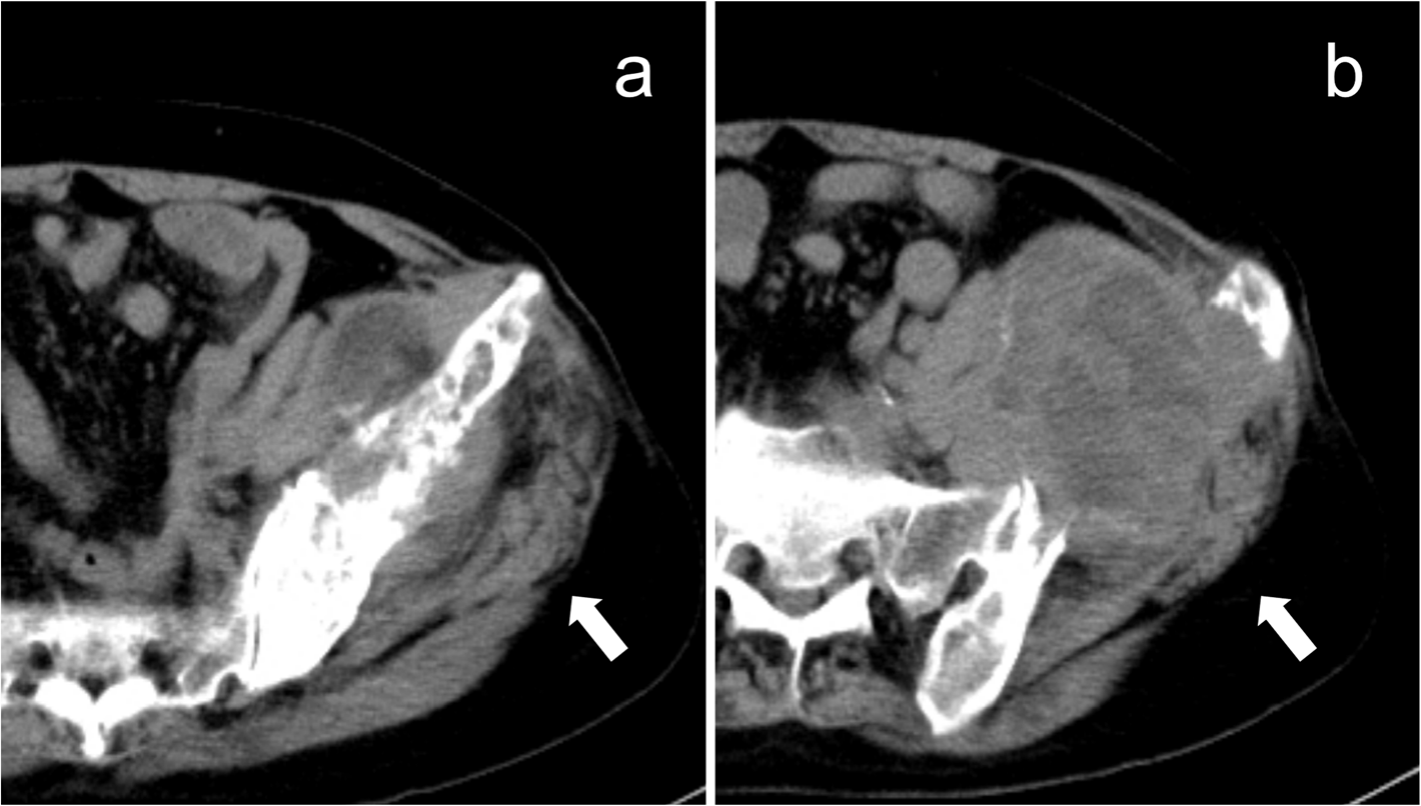
CT scan of the pelvis. **a** Expansive osteolytic bone destruction on the left iliac wing involving the acetabulum. Bone cement fillings are shown inside (white arrows). **b** Irregular soft tissue mass, about 9.5 × 8.4 × 7.6 cm on the left bone destruction area and its medial surface, with uneven density, and patchy low-density is visible inside (white arrow)

Computerized tomography of the pelvis showed a 9.5 × 8.4 × 7.6 cm uneven mass in the bone destruction area protruding into the pelvic cavity and compressing the left internal iliac and psoas muscles (Fig. 2a, b). The left iliac wing osteolytic bone was destroyed and the acetabulum was affected.

**Fig. 3:**
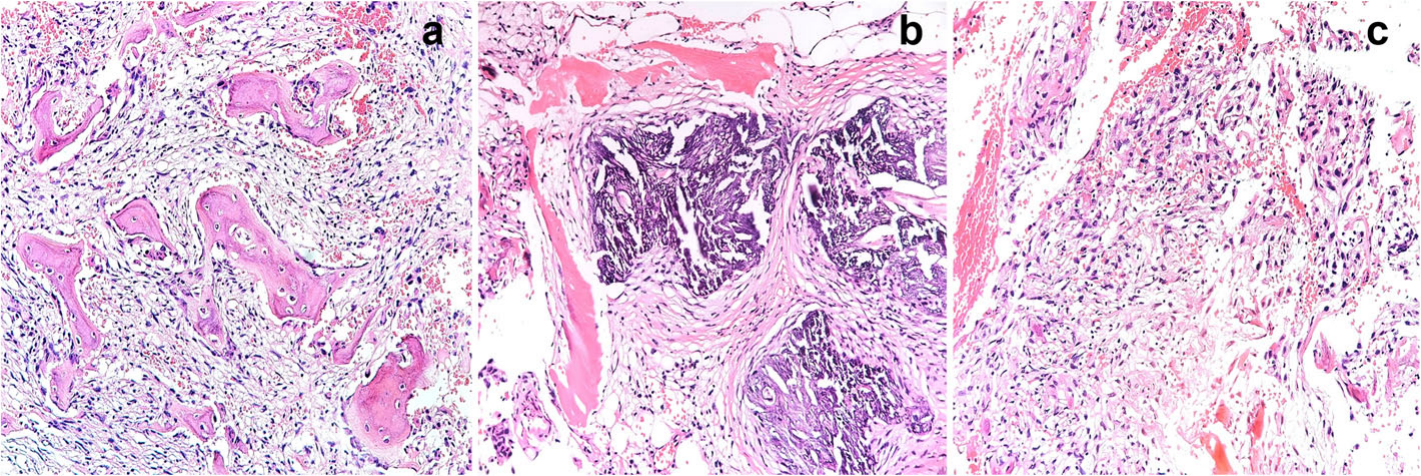
First surgical specimen. **a** Ossifying fibroma-like area: It is formed by the proliferation of mild spindle cells, accompanied by new bone trabeculae lined by osteoblasts. **b** Blue-purple smoky calcification can be seen around the remaining host bone. **c** The most actively proliferating spindle-shaped cell area. Some cells are slightly atypia showing no mitosis. (Haematoxylin and Eosin stained, original magnification × 100)

**Fig. 4:**
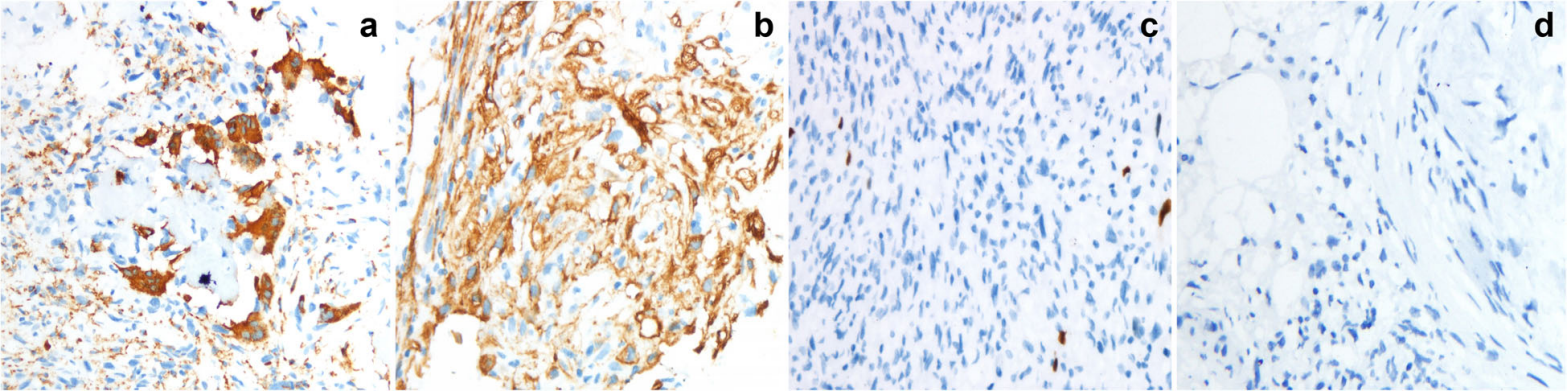
Immunohistochemistry. Tumor cells are immunopositive for CD68 (**a**), SMA (**b**), negative for Ki-67 (**c**, expression of<1%) and FGF-23 (**d**).(Immunohistochemical stained, original magnification × 400))

To further clarify the nature of the tumor, we observed the tumor tissue sections of the patient from 3 years ago. The histology at the time showed that the ossifying fibroma-like area of the tumor was mainly composed of mild spindle cells (Fig. 3a). A blue-purple smoky calcification could be seen around the remaining host bone (Fig. 3b). A small number of cells had slight atypia, without mitosis (Fig. 3c). Immunohistochemistry suggested the following: CD68+, SMA+, Ki67 expression of < 1%, FGF-23-CK-, S-100-, CD34-, P16-, EMA- (Fig. 4a-d). The patient’s initial diagnosis was PMT-ossifying fibroma-like recurrence of the left iliac bone.

**Fig. 5:**
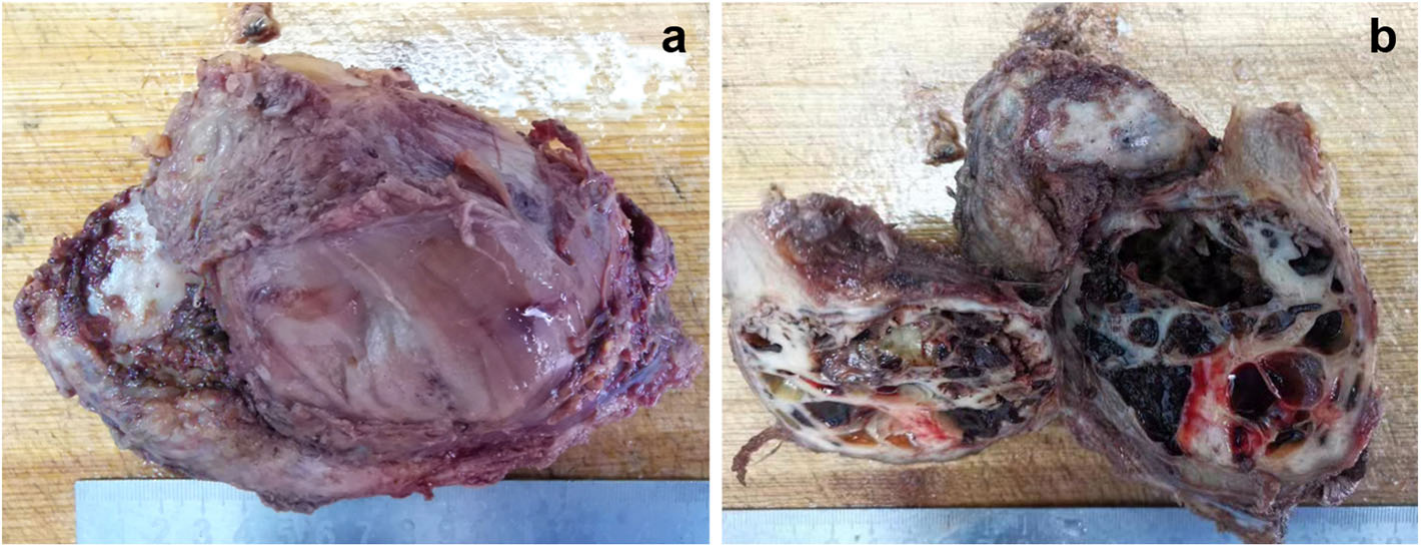
Left Ilium tumor. **a** The mass is 9 × 7 × 5 cm in size with the intact capsule. **b** The cut surface exhibited gray-brown-gray-white cysts, which contained gray and black blood clots with the rough inner wall, and the sac inner wall was rough. The sac wall thickness was about 0.1–1.0 cm

According to the surgical plan, the patient underwent left iliac wing tumor resection, pelvic bone grafting, and plate internal fixation. After successful anesthesia, the patient was positioned on her right side, a herringbone incision was made with the lateral iliac crest of the left iliac bone as the highest point, and a herringbone incision was made. The anterior incision extended to the midpoint of the inguinal ligament, extending outside. We used an electric knife to make an incision along the medial and lateral subperiosteal of the iliac bone, and gradually peeled off the muscles toward the deep surface without cutting the iliopsoas and sartorius muscles, and peeled each muscle from the attachment point of the pelvis one by one to expose the iliac wings. The dissection range was about 4 cm from the tumor. Osteotomy was used to cut the anterior superior iliac spine and the iliac bone about 1 cm above the sacroiliac joint. All the left iliac wing and the tumor were removed and sent for pathological examination. The hand field was then soaked in sterilized distilled water to stop local bleeding. An appropriate size of the artificial bone block was selected and pressed on the remaining ilium osteotomy screen, two steel plates were placed and fixed with screws. The C-arm fluoroscopy and internal fixation were excellent. During the operation, we found that the left iliac wing was extensively invaded by the tumor. The tumor traversed the iliac wing, the size was about 10 × 10 cm, the capsule was intact, and nourishing blood vessels were growing into it. The structure of the left iliac bone was destroyed, and tumor-infiltrating growth was observed in the bone (Fig. 5a, b).

**Fig. 6:**
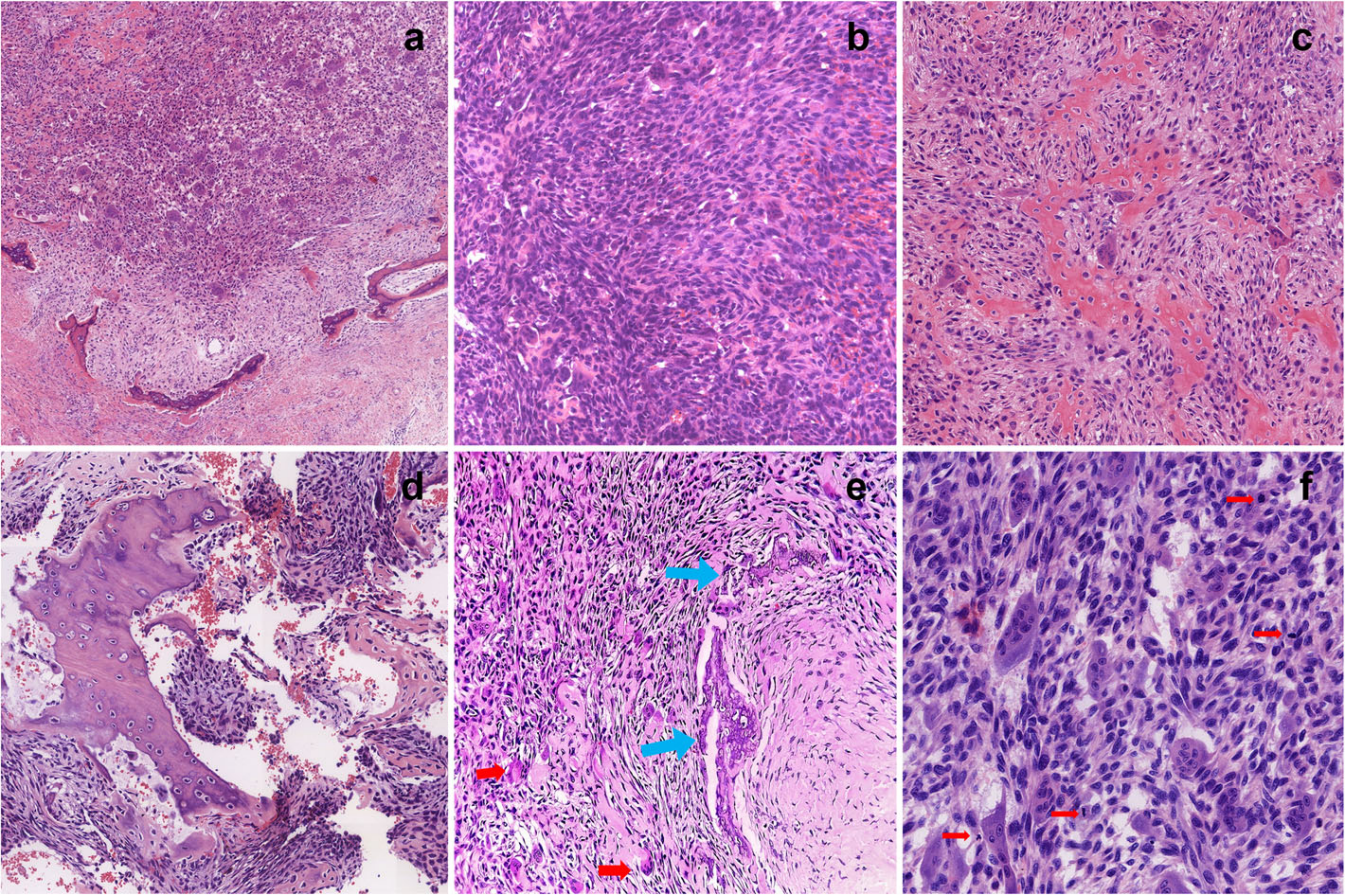
Left ilium tumor removal operation. **a** Infiltrative tumor borders, incomplete “bone shell”-like structures around the tumor (Haematoxylin and Eosin stained, original magnification× 100). **b** The tumor cell is oval, spindle, or star-shaped, has a small nucleus and nucleolus, has increased density, and has a fibrous histiocytoma-like structure (Haematoxylin and Eosin stained, original magnification× 200). **c** A small amount of ossifying fibroma-like areas can still be seen in recurrent tumors, the cell density is increased, the cells are mild to moderately atypia, and mitosis is easily seen (Haematoxylin and Eosin stained, original magnification× 200). **d** Tumor infiltration destroys the surrounding host bones and smoke-like calcification is not obvious (Haematoxylin and Eosin stained, original magnification× 200). **e** Occasionally smoked calcifications (blue arrows) and scattered osteoclast-like giant cells (red arrows) are seen in recurrent tumors (Haematoxylin and Eosin stained, original magnification× 200). **f** The mitotic figures (red arrow) have more than 5/10 HPF and moderate dysplasia is seen (Haematoxylin and Eosin stained, original magnification× 400)

**Fig. 7:**
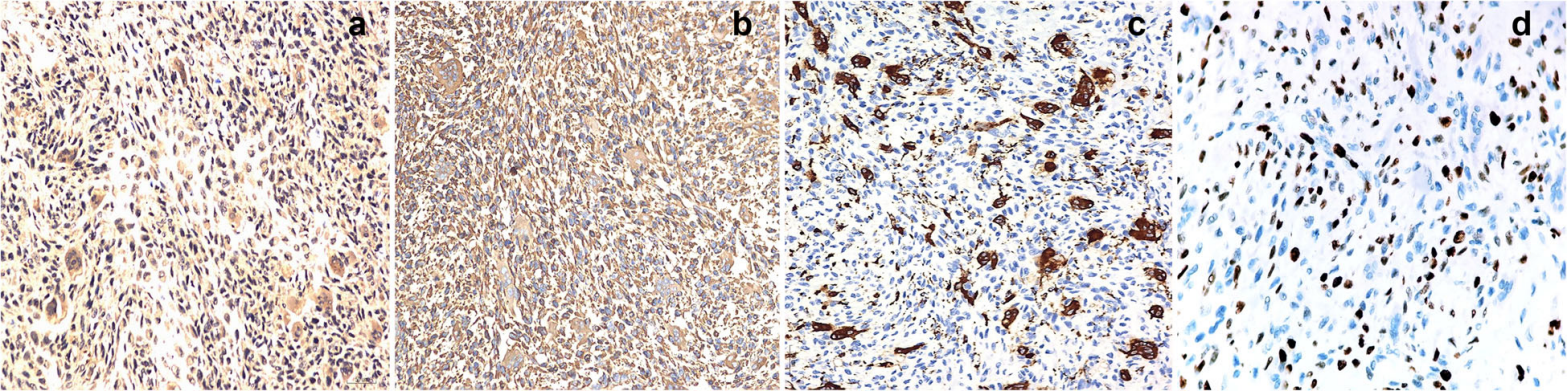
Immunohistochemistry. Tumor cells are immunopositive for FGF-23 (**a**), Vimentin (**b**), CD68 (**c**) and Ki-67 > 20% (**d**). (Immunohistochemical stained, original magnification× 400)

**Fig. 8:**
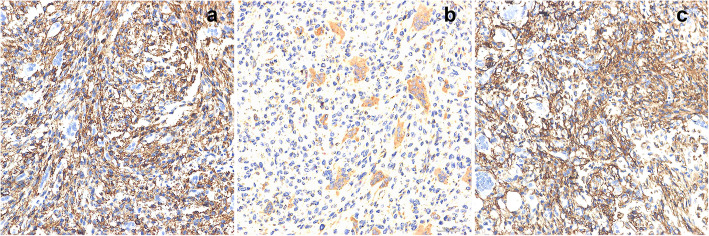
Immunohistochemistry. Tumour cells are immunopositive for SMA (**a**), Bcl-2 (**b**) and CD56 (**c**). (Immunohistochemical stained, original magnification× 400)

**Fig. 9:**
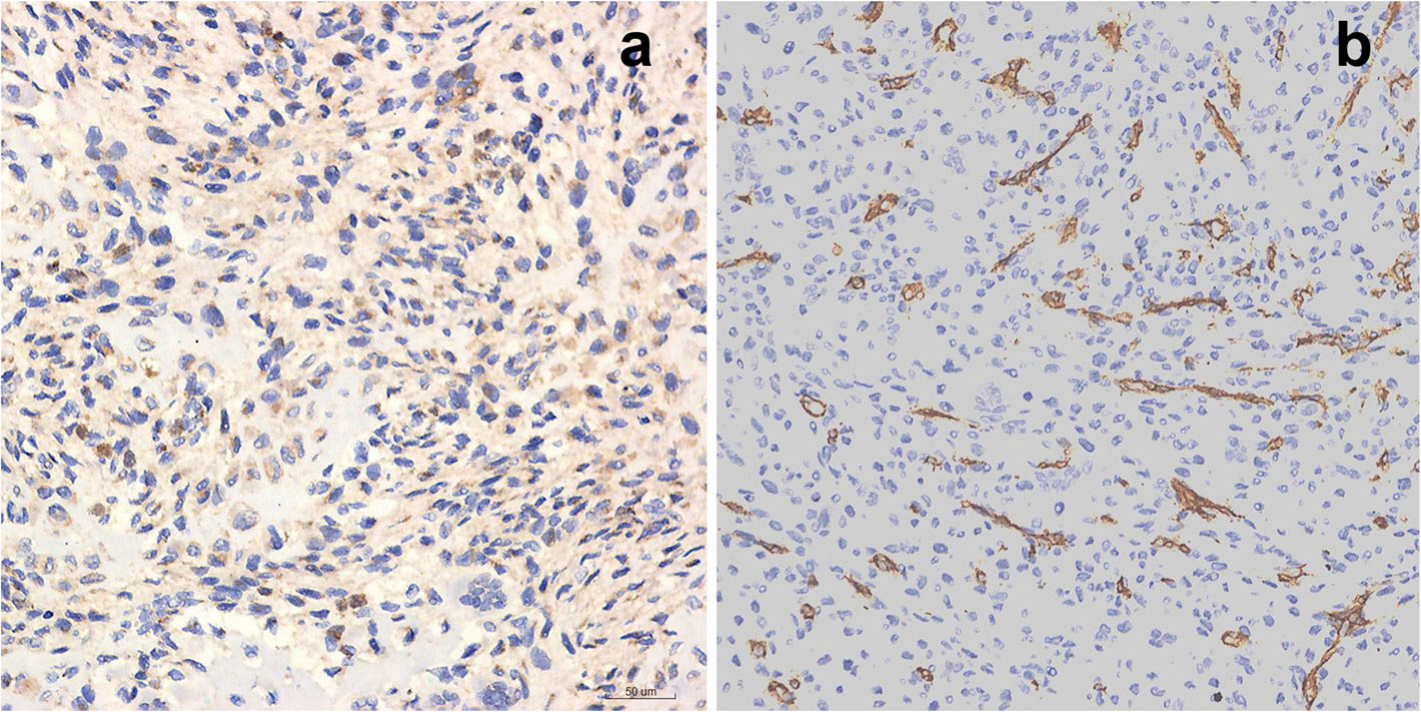
Immunohistochemistry. Tumour cells are immunopositive for CD99 (weak) (**a**) but negative for CD34 (**b**). (Immunohistochemical stained, original magnification× 400)

The histology of the patient’s tumor showed tumor cells were composed of oval, spindle, and star-shaped cells, with small nuclei and nucleoli, increased density, and a fibrous histiocytoma-like structure (Fig. 6a, b). Tumor invasion destroyed the surrounding host bones (Fig. 6d). There was an osteoclast-like multinucleated giant cell response showing hemosiderin deposits (Fig. 6e) and bone-like matrix formation (Fig. 6c). Smoke-like calcification was not obvious (Fig. 6e). The mitotic figures have more than 5/10 high-power fields (HPFs) and moderate dysplasia is seen (Fig. 6F). Immunohistochemistry of specimen suggested the following: FGF-23+, Vimentin+, CD68+, Ki67 > 20% (Fig. 7a-d), SMA+, Bcl-2+, CD56 + (Fig. 8a-c), CD99+, CD34- (Fig. 9a, b).

**Fig. 10:**
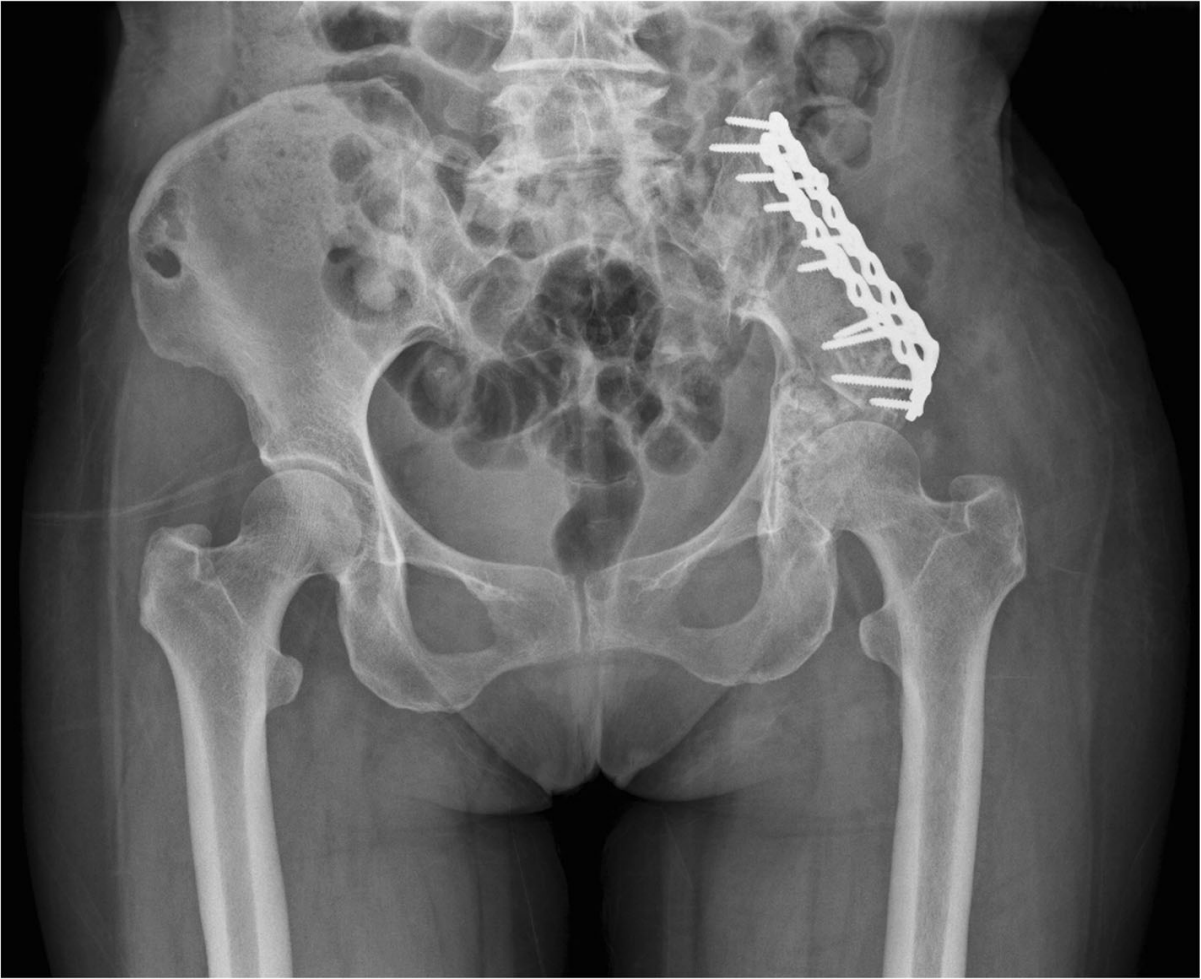
X-ray of pelvis. The left iliac bone showed postoperative changes. The plate and screws were well fixed. No tumor remained or recurred. There was no abnormality in the remaining bone of the pelvis, and no abnormality in the remaining soft tissues around the pelvis

Based on the histopathology and immunohistochemistry of the two surgical specimens, the pathological diagnosis of a recurring malignant left ilium PMT-ossifying fibroma-like subtype was confirmed. Post-surgery, the patient’s left hip soreness improved and the alkaline phosphatase levels returned to normal (Table 1). The patient came for a follow-up examination 3 months after the operation. During this period, she did not have pain in the left hip, the hip joints on both sides moved well, and she was able to walk normally. Re-examination of the pelvic X-ray (Fig. 10) showed that there was no residual tumor or recurrence, and the plate was well fixed. The serum phosphorus, serum calcium and serum alkaline phosphatase levels were all normal (Table 1). We then followed the patient by telephone for nearly 15 months. During this period, the left hip pain did not reappear and there was no tumor recurrence.

**Table 1:** Laboratory test results before and after tumor resection

Parameter	Before surgery	1 day after surgery	4 days after surgery	3 months after surgery	15 months after surgery
Phosphorus (0.9–1.34 mmol/L)	1.150 mmol/L	0.950 mmol/L	1.080 mmol/L	1.113 mmol/L	1.125 mmol/L
ALP(50-135 U/L)	211 U/L **↑**	81 U/L	60 U/L	65 U/L	72 U/L
Calcium (2.25–2.75 mmol/L)	2.510 mmol/L	1.840 mmol/L↓	2.080 mmol/L	2.450 mmol/L	2.635 mmol/L

Phosphorus: serum phosphorus; *ALP* Alkaline phosphatase; Calcium: serum calcium; **↑**: above normal levels;↓:below normal levels

## Discussion and conclusions

Herein we reported the clinical manifestations, histological, and pathological features of a 57-year-old Chinese woman with recurrent and malignant transformation of an ossifying fibroma-like PMT in the left iliac bone. She was successfully treated and did not show recurrent manifestations in the 15-month follow-up period.

PMT is a rare interstitial tumor. The main clinical manifestations are osteomalacia, hypophosphatemia, hematuria and neoplastic lesions [2]. The disease mostly occurs in middle-aged people, and there is no gender advantage [5]. It can occur in the bones and soft tissues of the whole body. According to reports, approximately 53% of PMT occurs in bones, 45% in soft tissues, and 3% in the skin. The most common complication is observed in the limbs, especially the lower limbs, followed by the head and neck [6]. A retrospective analysis of 39 cases of diabetes showed that 56% (22/39) of the tumors are located in lower limbs, 5% (2/39) in the upper limbs, 3% (1/39) in the hip joint, 31% (12/39) in the head (eight of which in the mandible and maxillary sinuses, and four in the sinuses), and 5% (2/39) in the chest [7].

In clinical practice, Honda et al. found that patients with PMT are usually associated with a history of osteomalacia, and the patients showed typical features of osteomalacia in the early stage, including generalized fatigue, bone pain, musculoskeletal weakness and incomplete fractures. The clinical manifestations include hypophosphatemia and decreased serum 1,25(OH)2-vitamin D3 levels [8]. Detection of serum or tumor tissue fibroblast growth factor 23 (FGF-23) has a subsidiary value in PMT diagnosis [9]. FGF-23 is a hormone-like protein secreted by PMTs. It reduces the level of 1,25-(OH)-D3 and inhibits 1-α-hydroxylase levels and the transport and reabsorption of phosphate in the proximal renal tubules, thus increasing urinary phosphate levels. Overall, FGF-23 activity leads to the loss of calcium and phosphate in the bones as well as reduced osteogenic activity, which could be a contributing factor to PMT-related osteomalacia [10]. However, not all patients with PMT have tumor-induced osteomalacia (TIO) symptoms. It is reported in the literature that about 80% of patients suffer from TIO [11]. The immunohistochemical expression of FGF23 is highly specific to PMT with TIO and can be used for a conclusive diagnosis of PMT. However, this does not apply to PMT without TIO [12]. The reason for the absence of TIO symptoms may be that the FGF-23 levels secreted by the tumor are not enough to cause TIO and change serum phosphorus levels [3]. In the present case, the patient had no obvious TIO, and serum calcium and phosphorus levels were normal. This might be due to insufficient FGF-23 secretion by the tumor, or there might be other compensation mechanisms. Because FGF-23 detection is not included in the routine testing in our hospital, this patient had no serum FGF-23 results. A previous retrospective analysis of 144 cases showed that the median FGF23 level for benign PMT-MCT is 302.9 pg/ml (range 42.6 to 706.5) [13]. In contrast, serum FGF23 level in adult female patients at the time of diagnosis of malignant PMT-MCT can reach up to 3319 ng/ml (normal reference value 10–50 pg/ml) [14].

In 1987, Weidner [1] first described the histological morphology of PMT and divided it into four histological subtypes: mixed connective tissue, osteoblastoma-like, non-ossifying fibroma-like, and ossifying fibroma-like [1, 2]. Among the 204 cases of PMT reported by Weidner, there are 3 cases of ossified fibromatous subtype, accounting for about 1%, and 13 cases of malignant PMT, accounting for about 6%.Mixed connective tissue subtypes account for almost 90% of PMT [1]. Our case is the first case report of PMT of the malignant ossifying fibromatoid subtype. The histological morphology of mixed connective tissue subtype PMT is mainly composed of blood vessels, mild spindle, or stellate cells without atypia. These cells are stuck in a mucous-like or mucous and cartilage-like matrix and usually have a bloody hemangioendothelioma-like structure. Furthermore, another typical feature is the smoke-like matrix produced by tumor cells, which is unusually flocculent, villi-like, or dirty after calcification [3, 15]. The microscopic characteristics of an ossifying fibroma-like subtype is tumor cells composed of spindle cells or spindle cell bundles, organized in a bundle and star shape. Osteoclast-like giant cells and osteoid regions are interspersed between the spindle or spindle cells, and the blood vessel development is moderate. The nuclei are a little atypical, with 1 or 2 mitotic maps per 10 HPF [1]. Due to the morphological diversity of PMT, it may be easily misdiagnosed. A further difficulty may be attributed to most PMTs being morphologically benign and slow-growing to detect malignant transformation. Clinical manifestations of malignant PMTs include local recurrence or distant metastasis with heterotrophic spindle cells displaying hyperplasia and increased mitotic figures (> 5/10 HPF) with sarcoma-like morphology and Ki67 expression of > 10% [16].

In most cases, tumor excision can alleviate PMT clinical symptoms and restore the levels of biochemical indicators [16]. In cases where the PMT site is not clear or the tumor cannot be completely removed, supplementation with calcium, phosphate, and active vitamin D can help manage the symptoms [17]. However, drug treatment is ineffective in the long run and may cause related complications, such as hyperparathyroidism, hypercalcemia, and kidney stone formation [3, 18]. Radical radiotherapy is the main treatment option for unresectable tumors and incomplete removal of remnants [16, 19, 20].

PMT is a rare and unique mesenchymal tumor with heterogeneous but recognizable histology. Due to the increased secretion of FGF23, it often causes clinical paraneoplastic syndromes, which consist of hypophosphatemic hyperphosphorylation osteomalacia. Patients usually show progressive muscle weakness, bone pain, and pathological fractures. Due to the non-specific nature of these symptoms, the lack of clinical suspicion, the failure to include serum phosphorus levels in routine blood chemistry tests and the difficulty in determining the responsible tumor, the diagnosis is usually delayed for several years. In addition, due to the rarity of these tumors and the morphological overlap with other mesenchymal tumors, they are often missed in histology.

## Data Availability

All data generated or analyzed during this study are included in this published article [and its supplementary information files].

## Abbreviations

PMT: Phosphaturic mesenchymal tumor
HPFs: High-power fields
1,25-(OH)-D3: 1-α,25-dihydroxyvitamin D3
FGF-23: Fibroblast Growth Factor
TIO: Tumor-induced osteomalacia

## References

[1] Weidner N, Santa CD (1987). Phosphaturic mesenchymal tumors. A polymorphous group causing osteomalacia or rickets. Cancer.

[2] Folpe AL, Fanburg-Smith JC, Billings SD, Bisceglia M, Bertoni F, Cho JY (2004). Most osteomalacia-associated mesenchymal tumors are a single histopathologic entity: an analysis of 32 cases and a comprehensive review of the literature. Am J Surg Pathol.

[3] Jan de Beur SM (2005). Tumor-induced osteomalacia. Jama.

[4] Yu WJ, He JW, Fu WZ, Wang C, Zhang ZL (2017). Reports of 17 Chinese patients with tumor-induced osteomalacia. J Bone Miner Metab.

[5] Ogose A, Hotta T, Emura I, Hatano H, Inoue Y, Umezu H (2001). Recurrent malignant variant of phosphaturic mesenchymal tumor with oncogenic osteomalacia. Skelet Radiol.

[6] Li D, Zhu R, Zhou L, Zhong D (2020). Clinical, histopathologic, subtype, and immunohistochemical analysis of jaw phosphaturic mesenchymal tumors. Medicine..

[7] Jiang Y, Xia W-B, Xing X-P, Silva BC, Li M, Wang O (2012). Tumor-induced osteomalacia: an important cause of adult-onset hypophosphatemic osteomalacia in China: report of 39 cases and review of the literature. J Bone Miner Res.

[8] Honda R, Kawabata Y, Ito S, Kikuchi F (2014). Phosphaturic mesenchymal tumor, mixed connective tissue type, non-phosphaturic variant: report of a case and review of 32 cases from the Japanese published work. J Dermatol.

[9] Qiu S, Cao LL, Qiu Y, Yan P, Li ZX, Du J (2017). Malignant phosphaturic mesenchymal tumor with pulmonary metastasis. Medicine.

[10] Savage CR, Zimmer LA (2009). Oncogenic osteomalacia from pterygopalatine fossa mass. J Laryngol Otol.

[11] Qiu S, Cao L-L, Qiu Y, Yan P, Li Z-x DJ (2017). Malignant phosphaturic mesenchymal tumor with pulmonary metastasis. Medicine..

[12] Shiba E, Matsuyama A, Shibuya R, Yabuki K, Harada H, Nakamoto M, et al. Immunohistochemical and molecular detection of the expression of FGF23 in phosphaturic mesenchymal tumors including the non-phosphaturic variant. Diagn Pathol. 2016;11:26. 10.1186/s13000-016-0477-3. 10.1186/s13000-016-0477-3 PMC4784377 26956379

[13] Oyama N, Kojima-Ishii K, Toda N, Matsuo T, Tocan V, Ohkubo K (2020). Malignant transformation of phosphaturic mesenchymal tumor: a case report and literature review. Clin Pediatr Endocrinol.

[14] Morimoto T, Takenaka S, Hashimoto N, Araki N, Myoui A, Yoshikawa H (2014). Malignant phosphaturic mesenchymal tumor of the pelvis: a report of two cases. Oncol Lett.

[15] Li D, Zhu R, Zhou L, Zhong D (2020). Clinical, histopathologic, subtype, and immunohistochemical analysis of jaw phosphaturic mesenchymal tumors. Medicine..

[16] Hautmann AH, Hautmann MG, Kolbl O, Herr W, Fleck M (2015). Tumor-induced Osteomalacia: an up-to-date review. Curr Rheumatol Rep.

[17] Yoshioka K, Nagata R, Ueda M, Yamaguchi T, Konishi Y, Hosoi M (2006). Phosphaturic mesenchymal tumor with symptoms related to osteomalacia that appeared one year after tumorectomy. Intern Med.

[18] Tang D, Wang XM, Zhang YS (2019). Oncogenic osteomalacia caused by a phosphaturic mesenchymal tumor of the femur: a case report. World J Clin Cases.

[19] Morimoto T, Takenaka S, Hashimoto N, Araki N, Myoui A, Yoshikawa H (2014). Malignant phosphaturic mesenchymal tumor of the pelvis: a report of two cases. Oncol Lett.

[20] Sidell D, Lai C, Bhuta S, Barnes L, Chhetri DK (2011). Malignant phosphaturic mesenchymal tumor of the larynx. Laryngoscope.

